# Supplementary material for: Cross-species screening of microsatellite markers for individual identification of snow petrel Pagodroma nivea and Wilson's storm petrel Oceanites oceanicus in Antarctica
Source: PeerJ. 2018 Jul 20;6:e5243. doi: 10.7717/peerj.5243 (PMC6055593; doi:10.7717/peerj.5243)
Supplement: Supplemental Information 5 — Results of Hardy-Weinberg equilibrium and Linkage disequilibrium tests for Wilson's storm petrel. The analyses was conducted using program Arlequin v 3.1. [file peerj-06-5243-s005.htm]

 Arlequin Result Browser - wsp-ARLEQUIN.arp
