# Supplementary figures and images for: Cross-species screening of microsatellite markers for individual identification of snow petrel Pagodroma nivea and Wilson's storm petrel Oceanites oceanicus in Antarctica
Source: PeerJ. 2018 Jul 20;6:e5243. doi: 10.7717/peerj.5243 (PMC6055593; doi:10.7717/peerj.5243)

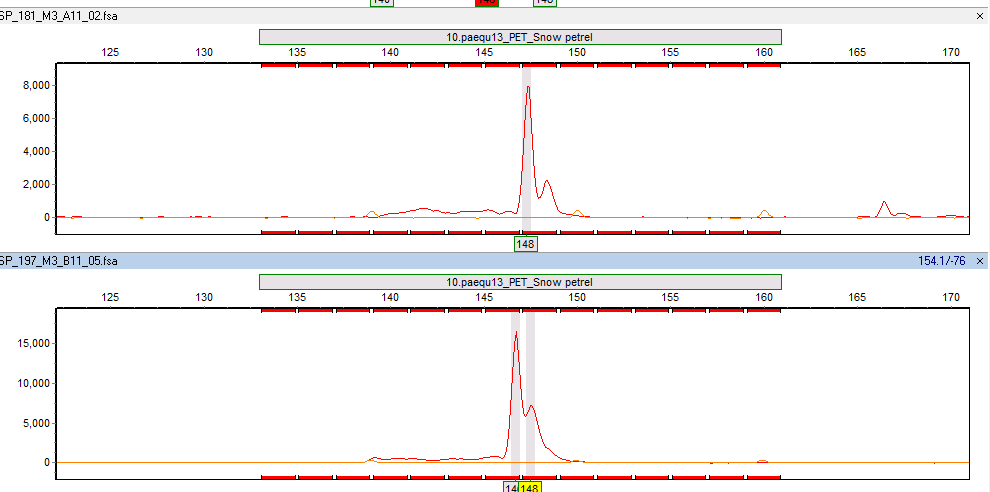


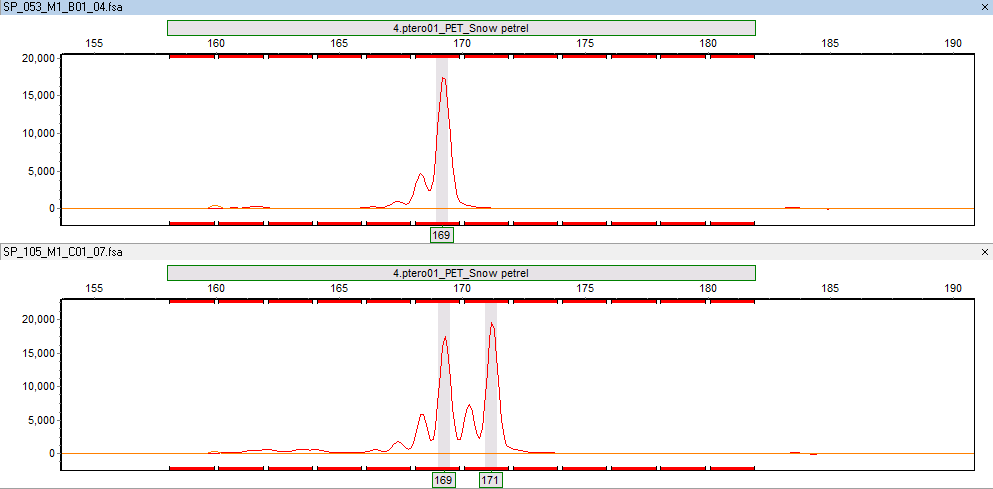


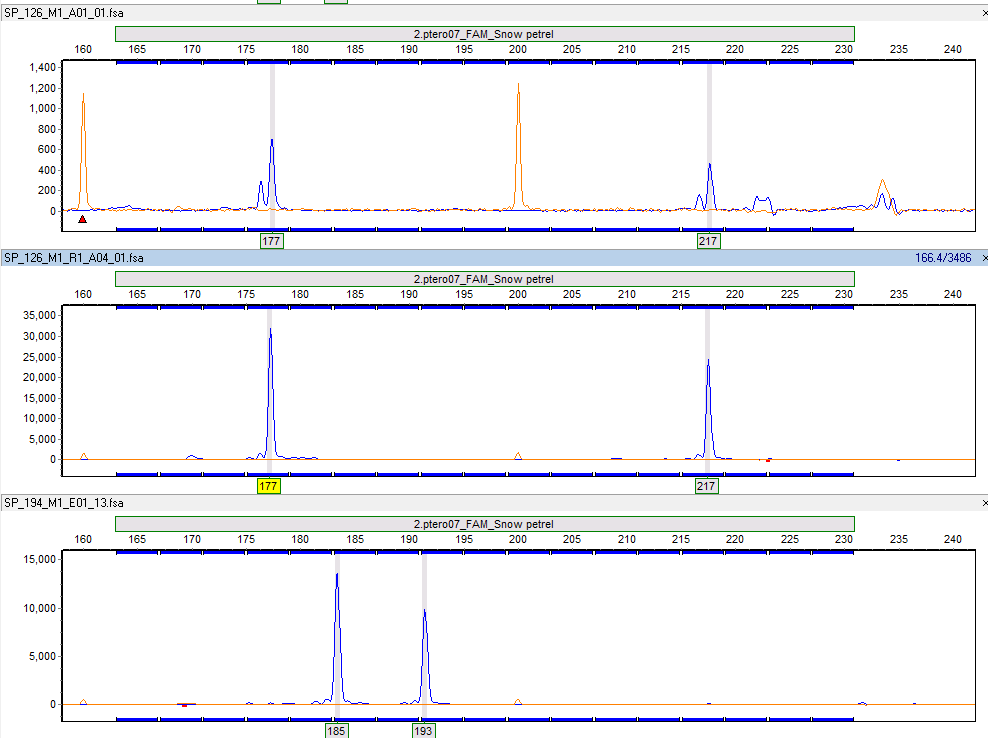


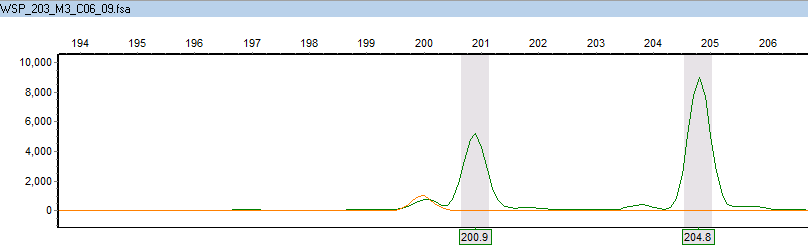

Supplement: Supplemental Information 7 — Examples of microsatellite electrophorograms for snow petrel and Wilson's storm petrel. [file peerj-06-5243-s007.docx]
